# OntoGene web services for biomedical text mining

**DOI:** 10.1186/1471-2105-15-S14-S6

**Published:** 2014-11-27

**Authors:** Fabio Rinaldi, Simon Clematide, Hernani Marques, Tilia Ellendorff, Martin Romacker, Raul Rodriguez-Esteban

**Affiliations:** 1 Institute of Computational Linguistics, University of Zurich, Binzmuhlestr. 14, 8050 Zurich, Switzerland; 2 pREDi (Pharma Research and Early Development Informatics), F. Hoffmann-La Roche Ltd, Grenzacherstrasse 124, 4070 Basel, Switzerland

**Keywords:** biomedical text mining, web services, OntoGene, BioC

## Abstract

Text mining services are rapidly becoming a crucial component of various knowledge management pipelines, for example in the process of database curation, or for exploration and enrichment of biomedical data within the pharmaceutical industry. Traditional architectures, based on monolithic applications, do not offer sufficient flexibility for a wide range of use case scenarios, and therefore open architectures, as provided by web services, are attracting increased interest.

We present an approach towards providing advanced text mining capabilities through web services, using a recently proposed standard for textual data interchange (BioC). The web services leverage a state-of-the-art platform for text mining (OntoGene) which has been tested in several community-organized evaluation challenges, with top ranked results in several of them.

## Background

Text mining technologies are increasingly providing an effective response to the growing demand for faster access to the vast amounts of information hidden in the literature. Several tools are becoming available which offer the capability to mine the literature for specific information, such as for example protein-protein interactions or drug-disease relationships. Examples of well known biomedical text mining tools are MetaMap [1], MedEvi [2], WhatIzIt [3], Gimli [4], iHOP [5], cTAKES [6], Open Biomedical Annotator [7].

The biomedical text mining community regularly verifies the progress of the field through competitive evaluations, such as BioCreative [8-10], BioNLP [11,12], i2b2 [13], CALBC [14], CLEF-ER [15], DDI [16], BioASQ [17], etc. Each of these competitions targets different aspects of the problem, sometimes with several sub-tasks, such as detection of mentions of specific entities (e.g. gene and chemicals), detection of protein interactions, assignment of Gene Ontology tags (BioCreative), detection of structured events (BioNLP), information extraction from clinical text (i2b2), large-scale entity detection (CALBC), multilingual entity detection (CLEF-ER), drug-drug interactions (DDI), question answering in biology (BioASQ).

There are numerous institutional attempts to structure some of the knowledge derived from the scientific literature into a more easily accessible format, such as that represented by life science databases. These are typically high-quality resources, where the primary data is accurately (and expensively) extracted from the scientific literature through a process of (mostly manual) curation. There are hundreds of disparate life sciences databases, each of which aims at representing as accurately as possible a particular subdomain. Examples of well-known biomedical databases are UniProt (proteins) [18], Entrez Gene (genes) [19], NCBI Taxonomy (species) [20], IntAct (protein interactions) [21], BioGrid (protein and genetic interactions) [22,23], PharmGKB (drug-gene-disease relations) [24], CTD (chemical-gene-disease relations) [25], and RegulonDB (regulatory interactions in *E. coli *) [26].

The OntoGene system is a text mining system which specializes in the detection of entities and relationships from selected categories, such as proteins, genes, drugs, diseases, chemicals. OntoGene derives its lexical resources from life sciences databases, thus allowing a deeper connection between the unstructured information contained in the literature and the structured information contained in databases. The quality of the system has been tested several times through participation in some of the community-organized evaluation campaigns, where it often obtained top-ranked results. One of the goals of the OntoGene group is to develop tools which support the process of curation of the biomedical literature, and promote a move towards *assisted curation*. By assisted curation we mean a combination of text mining approaches and the work of an expert curator, aimed at leveraging the power of text mining systems, while retaining the high quality associated with human expertise. We have implemented a platform for assisted curation called ODIN (OntoGene Document INspector) which aims at serving the needs of the curation community. The usage of ODIN as a tool for assisted curation has been tested within the scope of collaborations with curation groups, including PharmGKB [27], CTD [28], RegulonDB [29]. We believe that it is possible to gradually automate much of the most repetitive activities of the curation process, and therefore free up the creative resources of the curators for more challenging tasks, in order to enable a much more efficient and comprehensive curation process.

Assisted curation is also of utility in the process of pharmaceutical drug discovery. Many text mining tasks in drug discovery require both high precision and high recall, due to the importance of comprehensiveness and quality of the output. Text mining algorithms, however, cannot often achieve both high precision and high recall, sacrificing one for the other. Assisted curation can be paired with text mining algorithms which have high recall and moderate precision to produce results that are amenable to answer pharmaceutical problems with only a reasonable effort being allocated to curation.

In order to make the advanced text mining capabilities of the OntoGene system more widely accessible without the burden of installation of complex software, we have set up web services which allow any remote user to submit arbitrary documents. The results of the mining service (entities and relationships) are then delivered back to the user as XML data, or optionally, they can be inspected via a flexible web interface. There are strong drivers also in the pharmaceutical industry (for example at Hoffmann-La Roche) for the usage of web services for the annotation of free text. In particular, there is presently a strong trend to dissociate basic functionalities from siloed applications. It is a major advantage to have an open and modularized architecture where services can be combined into larger work-flows. The annotation web services provided by the OntoGene system fit exactly in this philosophy.

## Methods

The text mining pipeline which constitutes the core of the OntoGene system has been described previously in a number of publications [30-32]. We will only briefly describe the core text mining technologies, and instead focus mainly on the novel web services which allows remote access to the OntoGene text mining capabilities. One major recent modification, described in this paper, is the integration of a recently proposed standard for textual data interchange (BioC), which will be discussed later in this section.

The OntoGene text mining system contains modules for entity recognition and relation extraction, based on rule-based approaches (e.g. lexical lookup with variants) as well as machine-learning approaches (e.g. maximum entropy techniques). The first step in order to process a collection of biomedical literature consists in the annotation of names of relevant domain entities (currently the system considers proteins, genes, species, experimental methods, cell lines, chemicals, drugs and diseases). These names are sourced from reference databases and are associated with their unique identifiers in those databases, thus allowing resolution of synonyms and cross-linking among different resources.

One of the problems with sourcing resources from several databases is the possible inconsistencies among them. The fact that domain knowledge is scattered across dozens of data sources, occasionally also with some incompatibilities among them, is a severe problem in the life sciences. Ideally these resources should be integrated in a single repository, as some projects are attempting to do (e.g. OpenPhacts [33]), allowing querying within an unified platform. However, a deep integration of the information provided by the scientific literature and the content of the databases is still missing.

We train our system using the knowledge provided by life sciences databases as our gold standard, instead of hand-labeled corpora, since we believe that the scope and size of manually annotated corpora, however much effort has been invested in creating them, is not sufficient to capture the wide variety of linguistic phenomena that can be encountered in the full corpus of biomedical literature, let alone other types of documents, such as internal scientific reports in the pharma industry, which are not represented at all in annotated corpora. For example, PubMed currently contains more than 23 million records, while the entire set of all annotated publications probably barely reaches a few thousand, most of them sparsely annotated for very specific purposes.

Our approach is related to other information extraction research. [34] uses the large semantic knowledge database Freebase for a *distant supervision *approach to optimize information extraction patterns. [35] used the FlyBase database and referenced PubMed abstracts to improve the recognition of gene names by machine learning. [36] presents early work on exploiting biomedical databases as weakly labeled training data to improve relation extraction. Similar techniques have been used very early in the OntoGene system. One example is a version of the system used for the participation of the OntoGene group in the 2006 BioCreative competitive evaluation of text mining systems [37]. For this competition, the OntoGene group generated a training set of positive and negative sentences using techniques of distant supervision. This training set was used for training a classifier able to distinguish between 'background' and 'novel' statements, i.e. sentences reporting previous work as opposed to sentences reporting the actual results generated by the experiment described in the paper. The results of the classifier were used to filter the output of the system and produce only interactions coming from sentences classified as "novel", since the setting of the challenge required the participants to deliver only the most relevant interactions mentioned in the paper. The applied method becomes clear in the following citation:*"A sentence is considered positive if it contains at least one pair of proteins belonging to one of the gold standard interactions for the abstract to which the sentence belongs" *[30]. Furthermore, a similar approach was also applied successfully in the version of the OntoGene system used for participation in the 2009 BioCreative competition [38], which obtained the best results among all participants in the extraction of protein-protein interactions from scientific literature [31].

The specific data source used for the process of training the system through distant supervision depends on the application for which OntoGene/ODIN was customized. For the BioCreative 2009 PPI challenge, the data was sourced from the IntAct database, and was used at various stages in the process: entity recognition, validation of the novel/background statement detection, validation of final PPI. In the more recent applications for the CTD database, CTD entities were used.

In OntoGene, a term normalization step is used to match the terms with their actual representation in the text, taking into account a number of possible surface variations. Our normalization rules are similar to the rules reported in [39-41]. This is followed by a disambiguation step which resolves the ambiguity of the matched terms [42]. This process is also used to generate the weakly labeled data for all subsequent training steps based on the distant supervision approach. A marked-up term can be ambiguous for three main reasons. First, a technical term can also be a word of the common language (e.g. a gene called folD, or a chemical called SEX: sodium ethyl xanthate). Although the capitalization in most cases should reveal that it is a special term, and not a common word, there are cases when proper capitalization is not respected. Second, the term can be assigned an ID from different term types. When for example GFP (*Green Fluorescent Protein*) is mentioned in text it is tempting to simply annotate it as an instance of a protein. In fact, most likely it is being mentioned because it is used as a component of an experimental method (fluorescence microscopy) and not for its biological role as a protein. This type of ambiguity is not very common, and we approach it using simple context-based rules that decide on the correct type assignment (e.g. experimental method rather than protein in the case of GFP), in a way similar to what was done in [43]. Third, the term can be assigned several IDs from a single type. This problem is widespread in particular for genes and proteins, which have very ambiguous names, due to the fact that the same gene or protein occurs in many different species. One way to disambiguate such protein names is to apply knowledge about the organisms that are most likely to be the focus of the experiments described in the articles. In [44], we have described an approach to create a ranked list of 'focus' organisms. We use such a list in the disambiguation process by removing all the IDs that do not correspond to one of the organisms detected as relevant for the paper. Additionally, the scores provided for each organism can be used in ranking the candidate IDs for each entity. Such a ranking is useful in a semi-automated curation environment where the curator is expected to take the final decision. However, it can also be used in a fully automated environment as a factor in computing a confidence value for any other derived information, such as interactions where the given entity participates.

**Figure 1:**
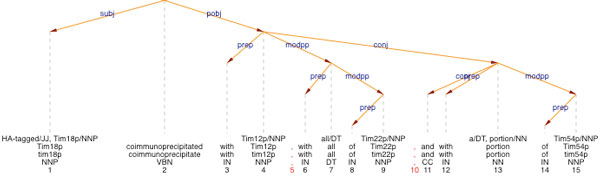
**Dependency parser output example**. Complete analysis of the sentence "*HA-tagged Tim18p coimmunoprecipitated with Tim12p, with all of Tim22p and with a portion of Tim54p*". The figure shows the linguistic processing performed by OntoGene: tokenization (each word is separated and assigned a unique identifier), part of speech tagging (assignment of grammatical category to each word, e.g. NN for noun), lemmatization (detection of grammatical root form, e.g. *coimmunoprecipitate*), and dependency-based syntactic analysis, represented in the figure by the tree-like orange arrows, which depict the syntactic structure of the sentences, with, for example, Tim18p as subject.

Candidate interactions are generated by simple co-occurence of entities within the same syntactic units. However, in order to increase precision, we parse the sentences with our state-of-the-art dependency parser [45], which generates a syntactic representation of the sentence. We have used an existing manually annotated corpus [46] as training corpus for the interaction detection task, based on an approach described in [47]. The corpus was parsed with our dependency parser which has been adapted to and evaluated on the biomedical domain [48,49]. The paths that are extracted from the corpus can directly be used for interaction detection. For example, in the sentence shown in Figure 1, a pattern with the decision 'yes' exists for the relation between *Tim18 *and *Tim12*, i.e. the pattern with top node *coimmunoprecipitate*, left path [subj] and right path [pobj]. The details of the algorithm are presented in [31]. The information delivered by the syntactic analysis is used as a factor in order to score and filter candidate interactions based on the syntactic fragment which connects the two participating entities. All available lexical and syntactic information is used in order to provide an optimized ranking for candidate interactions. The ranking of relation candidates is further optimized by a supervised machine learning method [50]. Since the term recognizer aims at high recall, it introduces several noisy concepts, which we want to automatically identify in order to penalize them. The goal is to identify some global preferences or biases which can be found in the reference database. One technique is to weight individual concepts according to their likeliness to appear as an entity in a correct relation, as seen in the target database.

**Figure 2:**
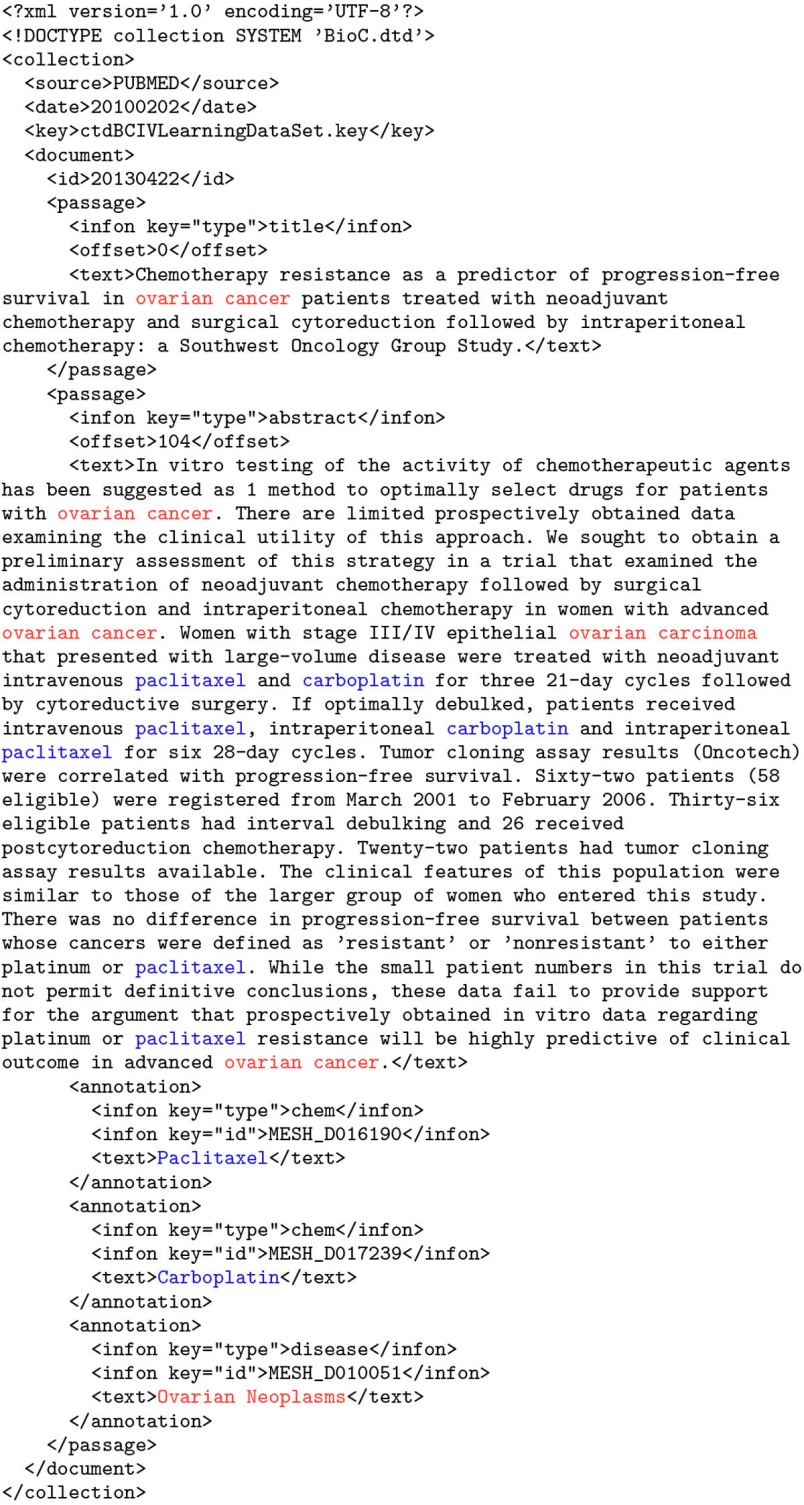
**Example of output of the OG text mining service in BioC format**. The output of the system is generated in the the BioC specification format. This output was generated by querying for chemicals and diseases on PubMed abstract 20130422. Colors added for clarity. Offsets of annotated terms can be obtained with a separate query.

The OntoGene web services have been implemented using the RESTful approach [51]. They accept simple XML files as input, based on the BioC specification. The output of the system is generated in the same format. For example, a query aiming at retrieving chemicals and diseases from PubMed abstract 20130422 would generate the output shown in Figure 2.

BioC [52] is a novel XML-based standard for data exchange and software integration specifically targeted at biomedical text mining systems. It was developed as an international collaboration of several leading text mining groups worldwide. BioC has the potential to contribute significantly to the advancement of text mining technologies for the biomedical literature, by enabling data sharing, allowing easier integration of different modules, and overcoming various technical barriers that so far have hindered the effective reuse of several tools contributed by the text mining community. BioC is a simple format for text representation which allows a precise but flexible annotation of entities mentioned in text and their relationships. Different implementations in various programming languages have recently been provided by the text mining community [53,54], which can be integrated as libraries in complex text mining applications, and offer tool developers the capability to deal with BioC annotations in a transparent fashion. Additionally, a significant number of corpora have been converted to BioC format, which will provide a critical mass for training future machine-learning based text mining systems.

BioC is a new annotation standard that has been spearheaded by the National Library of Medicine and researchers in the field of biomedical text mining. At present, the main alternatives to BioC in the biomedical realm are the GATE [55] and UIMA frameworks [56]. Each of these frameworks is built around a set of Java libraries that allows the interconnection of text mining components and the incorporation of external plug-ins, using an interoperability layer, to build text mining pipelines. Both UIMA and GATE, however, require a certain level of software expertise for their deployment and maintenance. BioC, on the other hand, provides a light, almost minimalist, alternative to UIMA and GATE by narrowing the scope of the framework to XML annotation standardization. Other XML annotation guidelines have been created for particular annotation types in the context of community challenges, such as BioCreative and the BioNLP Shared Tasks, or in the context of specific annotation tools. BioC has been created as a general standard that allows the flexibility of multiple types of annotations.

The OntoGene web services offer the user some options which can be used in the input query to select whether the result should contain in-line annotations (showing where exactly in the text the term was mentioned), or stand-off annotations (as in the example in Figure 2). Currently the system uses pre-defined terminology, and only allows the users to decide whether they want to use one of the pre-loaded vocabularies. However, we foresee in the future the possibility to upload user terminologies. Since the OntoGene system does not only deliver the specific terms found in the submitted articles, but also their unique identifiers in the source database(s), it is relatively easy to turn its results into a semantic representation, as long as the original databases are based on a standardized ontology. Any term annotation can be turned into a monadic ground fact (possibly using a suitable URI), and interactions can be turned into RDF statements, which could then potentially be integrated across a large collection of documents.

## Results

The OntoGene annotator offers an open architecture allowing for a considerable level of customization so that it is possible to plug in in-house terminologies. Besides the considerations at the level of the architecture, Roche has many different use cases that make use of annotation services. Text mining is applied to different text repositories or data feeds on a regular basis. These resources can be categorized into different classes. Open resources like PubMed or full text from PubMed Central are the starting point. Additionally, agreements with some editors permit full text mining from licensed journals [57]. Furthermore, data feeds which are again open (such as clinical trials) or commercial (like Adis R&D Insight, Pharmaprojects or Pharma Partnering) need to be mined. Finally, internal data - both free text and structured data - needs to considered. Given the variety of textual corpora that have to be dealt with, a toolbox of text mining services allowing customization and tailoring of a text mining pipeline to specific use cases is extremely welcome.

Users can submit arbitrary documents to the OntoGene mining services by embedding the text to be mined within a simple XML wrapper. Both input and output of the system are defined according to the BioC standard [52]. However, typical usage involves processing PubMed abstracts or PubMed Central full papers. In this case, the user can provide as input simply the PubMed identifier of the article. Optionally the user can specify which type of output they would like to obtain: if entities, which entity types, and if relationships, among which entity types.

**Figure 3:**
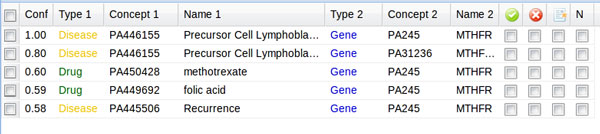
**ODIN interactions panel**. Example of interactions delivered by OntoGene as shown by ODIN. The confidence value for each candidate interaction is shown on the left. The two actors in the interactions are provided with their unique identifier, type, and reference name. The columns on the right allow the user to inspect and validate each candidate interaction.

The OntoGene pipeline identifies all relevant entities mentioned in the paper, and their interactions, and reports them back to the user as a ranked list (see Figure 3), where the ranking criteria is the system's own confidence for the specific result. The confidence value is computed taking into account several factors, including the relative frequency of the term in the article, its general frequency in PubMed, the context in which the term is mentioned, and the syntactic configuration between two interacting entities (for relationships). A detailed description of the factors that contribute to the computation of the confidence score can be found in [31].

**Figure 4:**
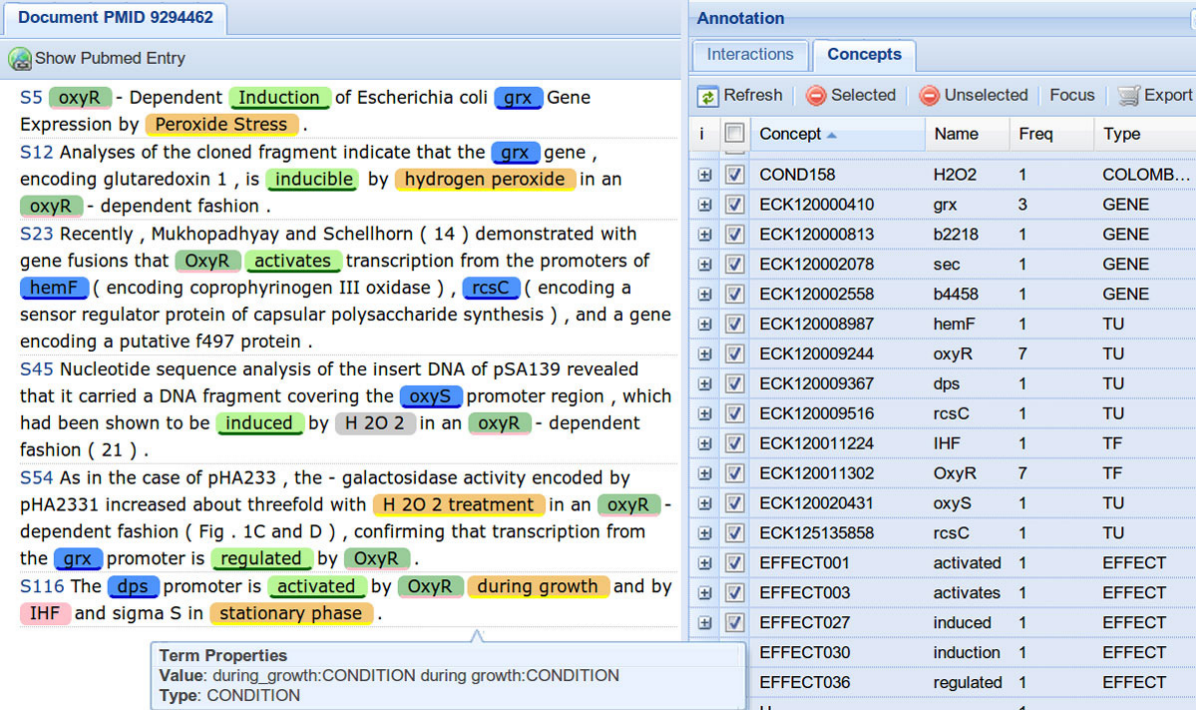
**ODIN screenshot**. Example of visualization of text mining results using the ODIN interface. The panel on the left shows the document with annotations, the panel on the right the corresponding concepts. The two panels are interconnected by the interface logic: whenever an item is selected in the concept panel, the corresponding terms are highlighted in the document panel.

The user can choose to either inspect the results, using the ODIN web interface (see Figure 4), or to have them delivered back via the RESTful web services in BioC XML format, for further local processing (see Figure 2). The set of sentences, individually enumerated, contained within those papers can then be viewed through ODIN (OntoGene Document Inspector), a flexible browser-based client application which interfaces with the OntoGene server. The curator can then use the features provided by ODIN to visualize selected annotations, together with the statements from which they were derived, and, if necessary, add, remove or modify them. Once the curator has validated a set of candidate annotations, they can be exported, using a standard format (e.g. CSV, RDF), for further processing by other tools, or for inclusion in a reference database, after a suitable format conversion.

In case of ambiguity, the curator is offered the opportunity to correct the choices made by the system, at any of the different levels of processing: entity identification and disambiguation, organism selection, interaction candidates. The curator can access all the possible readings given by the system and select the most accurate. Candidate interactions are presented in a ranked order, according to the score assigned by the system (see Figure 3). The curator can, for each of them, confirm, reject, or leave undecided. The results of the curation process can be fed back into the system, thus allowing incremental learning.

In order to allow the curators to focus on specific parts of the text that might be most relevant to their curation effort, the OntoGene team recently implemented a new capability within ODIN called "sentence filters", which allows users to specify a simple logical condition that functions as a filter, i.e. only sentences satisfying the specified condition are shown. In a recent experiment in collaboration with RegulonDB [58] such filters proved to be very effective, by allowing the curators to identify, from a subset of sentences constituting in average only 11% of the size of the original articles, all the information about regulatory interactions that they had originally found by reading the entire article set.

As a way to verify the quality of the core text mining functionalities of the OntoGene system, we have participated in a number of text mining evaluation campaigns [59,30,61]. Some of our most interesting results include best results in the detection of protein-protein interactions in BioCreative 2009 [31], top-ranked results in several tasks of BioCreative 2010 [62], best results in the triage task of BioCreative 2012 [59]. The usage of ODIN as a curation tool has been tested in collaborations with curation groups, including PharmGKB [63], CTD [27], RegulonDB [64]. Assisted curation is also one of the topics being evaluated at the BioCreative competitions [9,8], where OntoGene/ODIN participated with favorable results. The effectiveness of the web services has been recently evaluated within the scope of one of the BioCreative 2013 shared tasks [65]. Although different implementations can rapidly be produced upon request, a version of the system tailored for the Comparative Toxicogenomics Database (CTD) can currently be tested via the ODIN interface at the following URL:

http://www.ontogene.org/webservices/.

Since internally the original database identifiers are used to represent the entities and interactions detected by the system, the annotations can be easily converted into a semantic web format, by using a reference URI for each domain entity, and using RDF statements to express interactions. While it is possible to access the automatically generated annotations for further processing by a reasoner or integrator tool, we strongly believe that at present a process of semi-automated validation is preferable and would lead to better data consistency. A tool such as ODIN, which is currently used by database annotators for practical curation tasks, could also be put in the hands of authors which would be able to confirm or reject candidate annotations suggested by the system, thus allowing the construction of an enhanced document representation, integrating human readable text with machine readable semantic web statements.

The documents and the annotations are represented consistently within a single XML file, which also contains a detailed record log of the user interaction, which allows (if requested by the specific application) advanced analysis of user actions. The annotations are selectively presented, in an ergonomic way through CSS formatting, according to different view modalities. While the XML annotations are transparent to the annotator (who therefore does not need to have any specialized knowledge beyond biological expertise), his/her verification activities result in changes at the DOM of the XML document through client-side JavaScript. The use of modern AJAX technology allows for online integration of background information, e.g. information from different term and knowledge bases, or further integration of foreign text mining services.

The presence of the raw XML in the browser document gives the flexibility to compile dynamically tabular grid views of terms and relations including filtering, reordering, and editing the annotations in a spreadsheet-like way (this includes also chart visualizations). To keep the implementation effort feasible, the use of a dedicated JavaScript application framework is crucial. The advantage of a client-side presentation logic is the flexibility for the end user and the data transparency. For text mining applications, it is important to be able to link back curated metainformation to its textual evidence.

## Conclusion

We have presented novel web services which aim at making the text mining capabilities of the OntoGene system more easily accessible for a variety of potential users. OntoGene is a mature system which has been shown to perform at state-of-the-art level in a variety of text mining tasks. Open and modular web services, instead of monolithic applications, offer more flexibility to the end users, and are therefore a major advantage. However, such applications need to rely on simple and versatile standards for data exchange. We have discussed one of such standards, BioC, which is used within our text mining services.

Our system relies on dynamic adaptation of terminological resources from databases and ontologies, for named entity recognition and relation extraction. This approach can benefit from the large amount of knowledge present in curated biomedical databases to optimize and/or specialize text mining systems. Additionally, through the ODIN platform, we offer a sophisticated combination of automatic text mining and human curation and validation.

As a future development we envisage the possibility that ODIN could be turned into a tool for collaborative curation of the biomedical literature, with input from the text mining system aimed only at facilitating the curation process but not at fully replacing the knowledge of the human experts. Such social application could help address the widening gap between the amount of published literature and the capabilities of curation teams to keep abreast of it.

## Competing interests

The authors declare that they have no competing interests.

## Authors' contributions

FR conceived the work described in this paper and implemented parts of the system. SC implemented the initial versions of ODIN, and was responsible for the machine-learning approach aimed at optimizing ranking of the results. HM implemented the RESTful interface for the OntoGene web services. TE implemented some of the named entity recognition capabilities of the system. MR and RRE provided logistical support, use-case scenarios, and tested ODIN and the OntoGene web services.
